# Characterization and expression of the ABC family (G group) in ‘Dangshansuli’ pear (*Pyrus bretschneideri* Rehd.) and its russet mutant

**DOI:** 10.1590/1678-4685-GMB-2017-0109

**Published:** 2018

**Authors:** Zhaoqi Hou, Bing Jia, Fei Li, Pu Liu, Li Liu, Zhenfeng Ye, Liwu Zhu, Qi Wang, Wei Heng

**Affiliations:** 1 School of Horticulture, Anhui Agricultural University, Hefei, Anhui, P.R. China

**Keywords:** Pear, russet mutant, ABCG, evolution, gene expression

## Abstract

The plant genes encoding *ABCGs* that have been identified to date play a role in suberin formation in response to abiotic and biotic stress. In the present study, 80 *ABCG* genes were identified in ‘Dangshansuli’ Chinese white pear and designated as *PbABCGs*. Based on the structural characteristics and phylogenetic analysis, the *PbABCG* family genes could be classified into seven main groups: classes A-G. Segmental and dispersed duplications were the primary forces underlying the *PbABCG* gene family expansion in ‘Dangshansuli’ pear. Most of the *PbABCG* duplicated gene pairs date to the recent whole-genome duplication that occurred 30~45 million years ago. Purifying selection has also played a critical role in the evolution of the *ABCG* genes. Ten *PbABCG* genes screened in the transcriptome of ‘Dangshansuli’ pear and its russet mutant ‘Xiusu’ were validated, and the expression levels of the *PbABCG* genes exhibited significant differences at different stages. The results presented here will undoubtedly be useful for better understanding of the complexity of the *PbABCG* gene family and will facilitate the functional characterization of suberin formation in the russet mutant.

## Introduction

The ATP-binding cassette (ABC) superfamily includes a large and diverse group of proteins that play an important role in organ growth, plant nutrition, plant development, response to abiotic stress, and interaction of the plant with its environment. These transporters contain a highly conserved ATPase domain, the ABC (ATP-binding domain or nucleotide-binding domain, NBD), which hydrolyzes and binds ATP, supplying energy for the uptake of a variety of energy and for the extrusion of drugs and metabolic wastes from organelle and cells (Nicolás *et al.*, 2007).These subunits are encoded by individual genes (*ABCI* subfamily); by two genes, with each encoding one NBD and one TMD (half-size *ABC*s) that form heterodimers; by one gene encoding one NBD and one TMD (half-size) that form homodimers; or by a single gene (full-size *ABC*s) (Higgins and Linton, 2004). The subunits of the ABCA to ABCD proteins have a so-called forward TMD-NBD domain organization, whereas those of the ABCG subfamily are characterized by reverse NBD-TMD organization (Kang *et al.*, 2011).

ABC subfamily G (ABCG) includes both the half-size molecular transporter white-brown complex (WBC) and the full-size molecular transporter pleiotropic drug resistance (PDR). ABCG proteinis used in the production of a wide variety of substances (including antibiotics, prohormones, lignin monolignols, lipids and secondary metabolites) that are involved in many kinds of metabolic processes during the plant lifecycle. In Arabidopsis, the ABCG clade is the largest and includes both full-length and half-length transporters (Saha *et al.*, 2015). The ABCG subclass exhibits a TMD-NBD-TMD-NBD architecture and is divided into plant/fungal-specific pleiotropic drug resistance full-length transporters and eukaryotic white-brown complex half-size transporters that function as homo or heterodimers to create the TMD-NBD-TMD-NBD structure (Verrier *et al.*, 2008). The 28 half-size ABCG proteins compose the most complex ABC subclass, with diverse substrate specificity and various mechanisms required for dimerization for functionality.

Several plant ABCG proteins are known or suspected to contribute to the synthesis of extracellular barriers. *ABCG12*/*CER5* is required in the shoot epidermis for transporting lipid precursors for cutin and wax biosynthesis (McFarlane *et al.*, 2010). *ABCG11* is induced by salt, ABA, and wounding, and affects the expression of many genes implicated in cuticle metabolism and suberin formation in roots (Panikashvili *et al.*, 2010). *ABCG13* is closely related to *ABCG11* and *ABCG12* and contributes to cutin formation (Panikashvili *et al.*, 2011). *AtWBC11* is not only essential for developmental plasticity but also plays a vital role in stress responses (Panikashvili *et al.*, 2007). *ABCG15* plays a key role in the development of post-meiotic anthers and pollen exines in rice (Qin *et al.*, 2013). *ABCG29* exports monolignols required for lignin biosynthesis. Plant cuticular lipid export also requires ABC transporters (Alejandro *et al.*, 2012). *ABCG5* plays a role in the suberization of the hypodermis of rice roots, which contributes to the formation of the apoplastic barrier (Shiono *et al.*, 2014), and *ABCG1* is required for the formation of suberin in potato tuber periderm (Landgraf *et al.*, 2014). Moreover, *ABCG30* can increase phenolics and decrease sugars levels in Arabidopsis (Badri *et al.*, 2009). *ABCG2*, *ABCG6*, and *ABCG20* reduce the suberin load in seed coats but increase the suberin load in roots (Vishwanath *et al.*, 2015). *AtABCG22* can increase water transpiration and drought susceptibility (Kuromori *et al.*, 2011). Although functional divergence and high genetic redundancy hinder determination of ABC protein functions, some Arabidopsis ABCG family members are known to be involved in the export of cuticle components (Hofmann, 2014). At the same time, the ABCG subfamily has alsobeen observed in the plant response to abiotic stress through the transmembrane transport of hazardous materials or stress-related compounds. *AtABCG40* mediates the cellular uptake of the phytohormone abscisic acid (ABA), which is involved in the plant response to drought stress. In addition, AtABCG39, another ABCG protein from Arabidopsis, has been shown function as an importer in the cellular uptake of non-selective paraquat (Xi *et al.*, 2012).

A mutant branch of ‘Dangshansuli’ pear (*Pyrus bretschneideri* Rehd.) was investigated. The mature skin of ‘Dangshansuli’ pear is yellow-green, whereas the mutant is a russet color. To further explore the expression patterns of *PbABCG* family genes in ‘Dangshansuli’ and its russet mutant, ten *PbABCG* genes with differential expression (log_2_Ratio ≥ 1, FDR ≤ 0.001) were screened in the transcriptome of ‘Dangshansuli’ and its russet mutant (Heng *et al.*, 2016), which were validated at 25, 50, 75, 100, 125, 150, and 175 days after full bloom (DAFB) by real-time quantitative PCR (RT-qPCR), which would better reflect expression in different periods. The results will contribute to the understanding of the role of *PbABCGs* in the formation of suberin.

## Materials and Methods

### Identification of *PbABCG* genes in pear

The complete genome and proteome sequences and Gene-Finding Format (GFF) of Arabidopsis and pear were downloaded from the Arabidopsis Information Resource (version 10; http://www.arabidopsis.org) and http://peargenome.njau.edu.cn, respectively. In the proteome datasets, if two or more protein sequences at the same locus were identical where they overlapped, we selected the longer sequence. Two Hidden Markov Model (HMM) profiles for the ABC domains (PF00005) were downloaded from the Pfam protein family database (http://pfam.sanger.ac.uk/). HMMER (Eddy, 2011) was used to search a customized database containing the proteome, with a threshold set of at 1/100 the Pfam GA gathering cutoff. The HMMER-selected proteins were used for a BLASTP query of the original protein database. Finally, the BLASTP hits were scanned for ABCG domains using InterProScan.

### Chromosomal location and gene structure of *PbABCG*

The chromosome number is indicated on the chromosome. The synteny relationship between each pair of ABCG genes was detected using the MicroSyn software and positioned on the 17 pear chromosomes. The *PbABCG* gene names were assigned according to their position on pear chromosomes 1-17. The chromosome map showing the physical location of all *PbABCG* genes was generated with Circos software. Genes with a significant synteny relationship are connected by blue lines.

### Phylogenetic analysis of *PbABCG* genes

First, a neighbor-joining phylogenetic tree was created using the full-length protein sequences of ABCG from pear and Arabidopsis. Second, the starting point for our tree construction was the amino acid multiple sequence alignment created using MUSCLE with the default parameters (Edgar, 2004). An NJ tree was constructed using MEGA software using the Jones, Taylor and Thorton (JTT) model. A bootstrap analysis with 1,000 replicates was performed in each case by using the NJ method in MEGA (version 6.0) (Tamura *et al.*, 2013).

### Exon–intron structure and domain analysis

Exons, which are represented by boxes, were drawn to scale. Lines connecting two exons represent an intron. The PF00005 domain (the ATP-binding domain of ABC transporters) is marked in red. Intron phases 0, 1 and 2 are indicated by numbers 0, 1 and 2, respectively. The ABC domains were downloaded from the Pfam protein family database.

### Ks value and Ka/Ks ratio reveal dates and driving forces of evolution

MCScanX downstream analysis tools were used to annotate the Ka and Ks substitution rates of syntenic gene pairs. The mean Ks values of orthologous ABCG gene pairs between Chinese white pear and Arabidopsis were calculated using all homologous gene pairs located in the same synteny block. KaKs_Calculator 2.0 was used to determine the Ka and Ks (Wang *et al.*, 2010). To date the segmental duplication events, six consecutive homologous gene pairs on each side flanking the Hsf genes were chosen to calculate the mean Ks. For segments with fewer than 12 homologous genes, all available anchor pairs were used (Du *et al.*, 2013).

### Plant material

Fruit samples were collected at 25, 50, 75, 100, 125, 150 and 175 DAFB. The exocarps of ‘Dangshansuli’ (wild type, WT) and its russet mutant (mutant type, MT) from at least ten individual fruits were mixed at each stage, immediately frozen in liquid nitrogen and stored at -80°C until use. The samples at different stages were repeated three times. The exocarp was manually dissected from the fruit skin with a razor blade (0.5 mm thickness). The collected samples were immediately frozen in liquid nitrogen and stored at -80°C until RNA extraction.

### Expression analysis of *PbABCG* genes by RT-qPCR

The expression levels of differentially expressed genes were measured using RT-qPCR with SYBR green I chemistry. Gene-specific primer sequences (Table S2) were designed with the Primer Express software program and tested to ensure the successful amplification of single discrete bands and no primer-dimers.

Single-stranded cDNA was synthesized using an oligo (dT) primer (20-mer) by means of the High Capacity cDNA Reverse Transcription Kit (TakaraBiomedical Technology) using 2 μg of purified RNA. RT-qPCR was performed with the SYBR Green PCR Master Mix (TOYOBO (SHANGHAI) BIOTECH) and carried out in an optical 48-well plate using an ABI PRISM 7300 Sequence Detection System (Applied Biosystems). The reactions and profiles were run according to the methods of Heng *et al.* (2016). Three independent biological replicates were performed.

## Results

### Identification and classification of *PbABCG* genes in pear

To identify all potential *PbABCG* genes in ‘Dangshansuli’ pear, the ABCG protein domains were used in BLAST queries against the ‘Dangshansuli’ pear genome. As a result, 80 ABCG genes were identified (Table S1). According to the prediction, *PbABCG34h* is the gene encoding the longest sequence of amino acids, comprising approximately 1741, while the shortest is *PbABCG40i*, with only 164 amino acids. The positive (+) and negative (-) signs following each gene represent forward and reverse orientation of the respective gene. The molecular weights of these deduced PbABCG proteins ranged from 18.02 kDa (PbrABCG40i) to 196.94 kDa (PbrABCG34h), and the isoelectric points ranged from 6.24 (PbrABCG40i) to 9.97 (PbrABCG9) (Table S1).

The ABCG genes mapped onto the different chromosomes in the pear genome. Chr3 contained the most genes, whereas Chr1, Chr2, and Chr6 only have one gene. There were 13 genes in the scaffold with numerous contig stitching results. The gene position and size of each chromosome can be found on the top of each chromosome (Figure S1).The *PbABCG* genes identified in this study were distributed across the 17 chromosomes. The chromosome number is indicated on the top of each chromosome. Those genes are shown on the right of each chromosome. The black ring represents the centromere of each chromosome, and the dotted line indicates tandemly duplicated gene pairs.

### Localization and synteny of the *PbABCG* genes in pear

Circular visualization of the *PbABCG* genes was mapped onto the different chromosomes in the genome using Circos software. The *PbABCG* genes in ‘Dangshansuli’ pear were mapped onto the different chromosomes (Figure S2). The chromosome or scaffold number is indicated on the inner side, and short, red, highlighted lines in the inner circle correspond to different *PbABCG* genes. Gene pairs with a syntenic relationship are joined by a line. The *PbABCG* genes are distributed on the 17 pear chromosomes, with 80 *PbABCG* genes detected on all 17 chromosomes. Similar to that of the *PbABCG* genes, the distribution of the *PbABCG* genes on every chromosome is random (Figure S2).

### Phylogenetic tree of ABCG genes in pear and Arabidopsis

**Figure 1:**
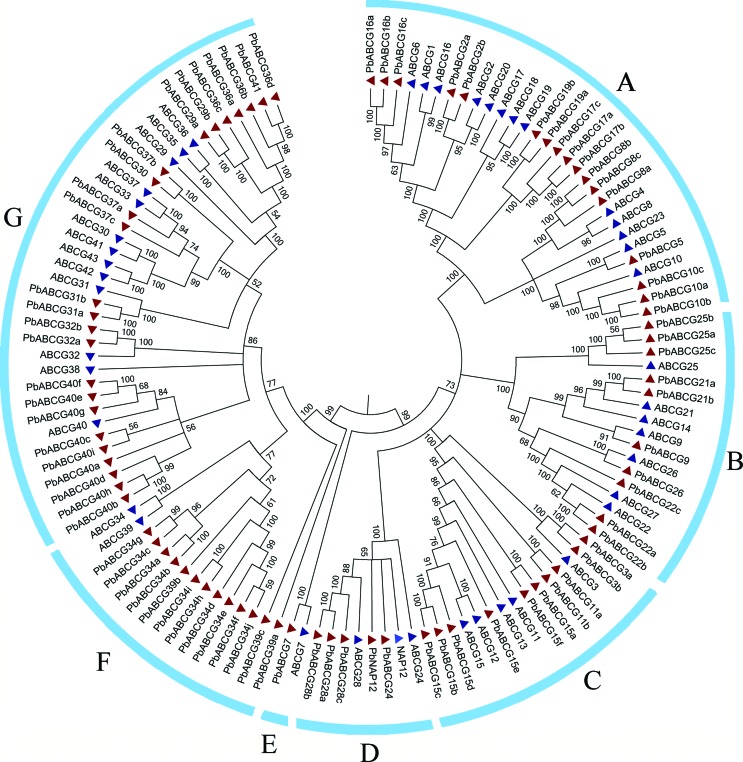
Phylogenetic tree of ABCG genes in pear and Arabidosis annotated with collinear and tandem relationships. The genes are divided into seven groups from A to G.

To determine the evolutionary relationships of the ABCG genes, an unrooted NJ phylogenetic tree using bootstrap analysis (1000 replicates) was constructed based on multiplesequence alignments of the 124 ABCG genes in pear and Arabidopsis. The 124 ABCG members could be classified into seven classes (A-G). Figure 1 shows the chain of genes, including *PbABCG21* and *AtABCG21*, most of which explain the Arabidopsis and pear homology. The chain also indicates that the function of the gene in pear may be similar to that in Arabidopsis. It was worth noting that many ABCG genes were classified as related sister pairs, including those in pear or Arabidopsis or in both, namely, *AtABCG1/AtABCG6*, *AtABCG2/AtABCG20*, *AtABCG8/AtABCG9*, *AtABCG29/AtABCG35/AtABCG36*, *AtABCG30/AtABCG33/AtABCG37/AtABCG41/AtABCG42/AtABCG43*, *AtABCG8/PbABCG8*, *AtABCG5/PbABCG5*, *AtABCG10/PbABCG10*, *AtABCG25/PbABCG25*, *AtABCG21/PbABCG21*, *AtABCG26/PbABCG26*, *AtABCG22/PbABCG22*, *AtABCG27/PbABCG27*, *AtABCG3/PbABCG3*, *AtABCG11/PbABCG11*, *AtABCG7/PbABCG7*, and *AtABCG32/PbABCG32*, all of which had a very strong bootstrap support (greater than 99%).

The results also indicated that *PbABCG34* and *AtABCG34* only had 77% support. *PbABCG40* and *AtABCG40* had 56% support. In pear, *PbABCG17* and *PbABCG19*, *PbABCG30* and *PbABCG37*, *PbABCG29* and *PbABCG35* and *PbABCG36* had a very strong bootstrap support (100%) (Figure 1). According to the classification criteria of Wu *et al.* (2013), the 124 ABCG genes were grouped into A-G classes. Class A contained 30 members, class B had 17 members, class C and D had 15 and 8 members, respectively, class E only had 2 members, and class F and G had 15 and 37 members, respectively. Interestingly, all classes contained ABCG members from pear and Arabidopsis. In the combined phylogenetic tree, there were a total of 19 sisters. The maximum number of sister pair members was found in group B, which had 11 sister members. In addition to the above 1:1 orthologous relationship, 1:n and n:1 orthologous relationships, such as those for the single *PbABCG29* and multiple *AtABCG36/29/35*, were also observed. The third n:n orthologous relationship was found in the class G cluster *AtABCG30/33/37/41/42/43* and for *PbABCG30/37* in class G. Moreover, *PbABCG37* and *PbABCG30*, *PbABCG29* and *PbABCG36*, and *PbABCG41* and *PbABCG36*, which constitute three genes of the chain, indicate that a certain correlation in the evolution and function of these three genes may exist.

### Conserved structural features and gene structure of ABCG in pear and Arabidopsis

The Multiple EM for Motif Elicitation (MEME) motif search tool was used to predict and verify domains in the PbABCG protein sequences. Twenty corresponding consensus motifs were detected (Figure S3). The numbers of motifs in the PbABCG protein sequences were quite variable. The members of the 80 genes contained the most conserved motifs, with the largest number (16) detected for *PbABCG34a*. *AtABCG12* possessed the fewest motifs, with only one intermediate number.

### Exon-intron compositions of pear ABCG genes

In general, the number of domains present in protein sequences is usually helpful for predicting the function of unknown genes, and a detailed analysis of the identified protein sequences is the first try to find useful clues about the roles of the corresponding ABCG genes. Most ABC transporters function as a dimer, and therefore are composed of four domains: two ABC modules and two TMDs. From Figure S4, one can see that all genes have PF00005 and that approximately 35 genes have a complete PF00005, and they do not have exon-intron compositions. According to Figure S4, the members of the ABCG gene family in Arabidopsis and pear contain 0-2 exons. However, the exon number in Class A was significantly less than in the other groups (Figure S4). We believe that the less conserved features of the DNA-binding domain resulted from a rapid divergence during evolution, which was likely the reason for the origin of different ABCG genes.

### Ks value and Ka/Ks ratio reveal dates and driving forces of evolution

The Ks value (synonymous substitutions per site) is widely used to estimate the evolutionary dates of whole genome duplication (WGD) or segmental duplication events. Genes within a single genome can be classified as singletons, dispersed duplicates, proximal duplicates, tandem duplicates or segmental/WGD duplicates depending on their copy number and genomic distribution. The Ks values in Arabidopsis and pear suggest two large-scale gene duplications. The main peak of Ks ranges from 0.15 to 0.3, whereas the secondary peak ranges from 1.5 to 1.8 (Wu *et al.*, 2013). In pear and apple, the recent WGD must have occurred at 30-45 MYA, while the ancient WGD must have resulted from an acknowledged paleohexaploidization event that occurred at ~140 MYA (Fawcett *et al.*, 2009). Protein amino acid sequences of all of the gene pairs were aligned and used to guide the alignments of DNA coding sequences (CDS). The syntenies between each pair of members were detected using MicroSyn software (Cai *et al.*, 2011). The parameters were as follows: window size of 100 genes, tandem gap value of 2, expected threshold value cutoff of 1e^-10^, and 8 homologous pairs to define a syntenic segment. The mean Ks values of orthologous gene pairs in the same synteny block and the Ka and Ks values were calculated by MicroSyn.

Based on the Ka and Ks values, only the function of *PbABCG28* was selected. Therefore, we used Ks values to estimate the evolutionary dates of the segmental duplication events among the *PbABCG* gene family. The mean Ks of the ABCG duplicated gene pairs in the syntenic region are shown in Table S3. The Ks values for the *PbABCG* gene pairs ranged from 0.04 to 2.38. We further inferred that the segmental duplications *PbABCG24* vs *PbNAP12* (Ks ~2.19), *PbABCG34b* vs *PbABCG34d* (Ks ~1.33), and *PbABCG37c* vs *PbABCG30* (Ks ~1.63) may have arisen from γ triplication (~140 MYA). Furthermore, many duplicated gene pairs had similar Ks values (0.21–0.32), suggesting that these duplications may have been derived from the same recent WGD (30~45 MYA). Surprisingly, two duplicated gene pairs (*PbABCG26* vs *PbNAP12* and *PbABCG16a* as well as *PbABCG2a* and *PbABCG16b* vs *PbABCG2a*) possessed higher Ks values (3.16~4.65), suggesting that these pairs might have arisen from a more ancient duplication event (Table S3).

### Expressions of 10 *PbABCG* genes in the exocarp of pear

**Figure 2:**
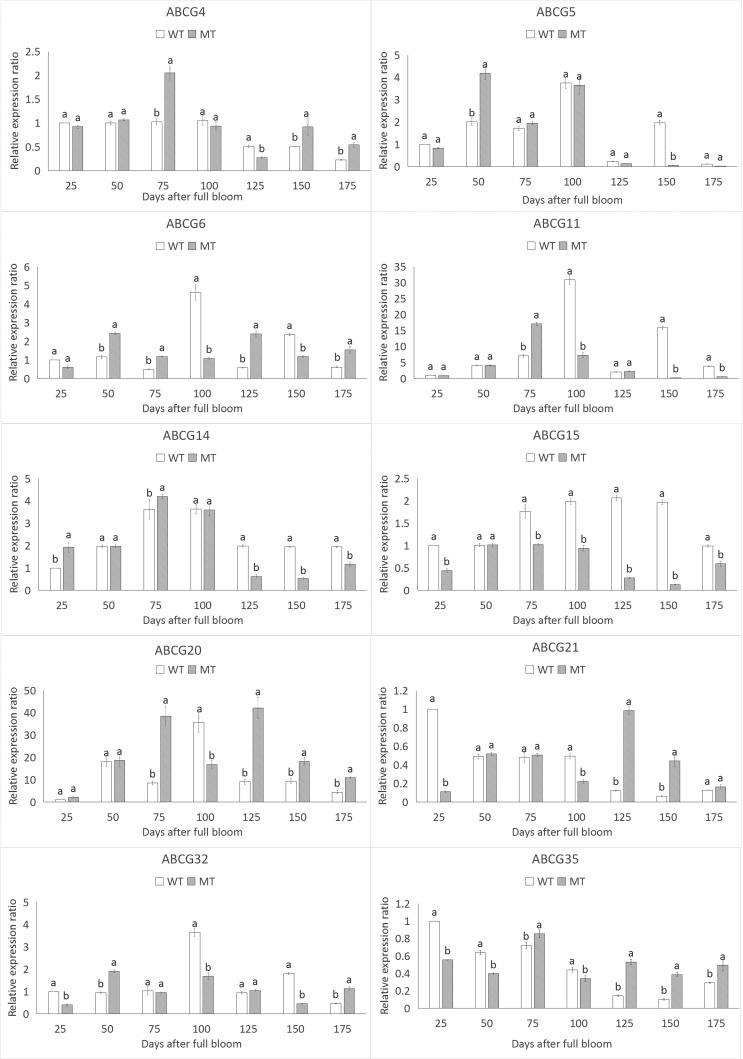
The relative expression levels of 10 *PbABCG* genes in the exocarps of ‘Dangshansuli’ and its russet mutant pear.

The expression of *PbABCG* genes was investigated at the transcriptional level, considering the increase in time in the exocarp of ‘Dangshansuli’ and ‘Xiusu’ pear. The CT values were used to measure the expression level of the *PbABCG* genes. This is because of the difficulty to analyze and compare proteins between ‘Dangshansuli’ pear and its russet mutant. Based on the results, we chose a maximum of 10 genes from the 80 genes to perform expression analyses. The expression patterns of the 10 *PbABCG* genes were very diverse, and most *PbABCG* genes exhibited some degree of stage specificity. Eight genes (*PbABCG4*, *PbABCG6*, *PbABCG11*, *PbABCG15*, *PbABCG20*, *PbABCG21*, *PbABCG32* and *PbABCG35*) were detected throughout fruit maturation. There was a significant difference between the two varieties in four stages (25~100 DAFB). Moreover, six *PbABCG* genes (*PbABCG4*, *PbABCG6*, *PbABCG11*, *PbABCG15*, *PbABCG20* and *PbABCG32*) showed increasing transcript levels with the passage of time, while *PbABCG21* and *PbABCG35* expression decreased with increasing time. However, *PbABCG6* and *PbABCG11* showed obvious differences in fruit development. In addition, the transcriptional changes of *PbABCG5* and *PbABCG14* were not clearly associated with time (Figure 2).

## Discussion

Members of the *PbABCG* gene family have been identified and analyzed in different land plant species. The number and composition of ABCG family members differ in various plants (Kang *et al.*, 2011). Ancient polyploidy events (also known as WGDs) and additional recent lineage-specific WGDs have presumably resulted in varying numbers of ABCG genes within flowering plants (Qiao *et al.*, 2015). Therefore, this recent WGD event likely led to the different numbers of *PbABCG* genes in the investigated pear species. Different patterns of gene duplication, such as genome-wide, tandem, and dispersed duplications, contribute differently to the expansion of specific gene families in plant genomes. Some large gene families, including the APETALA2/ethylene responsive element binding factor (AP2/ERF) and WRKY families, are more likely to expand by segmental and tandem duplications. These observations suggest that the expansion of these *PbABCG* genes occurred before the divergence of the Rosaceae species. Furthermore, the majority of the *PbABCG* genes were related more closely to *PtABCGs* than to *AtABCGs*. This result may be explained by the fact that *PtABCG* genes are associated with trees subjected to prolonged environmental stress.

The functional diversification of ABCG genes has been observed in several plant species. *ABCG1*, *ABCG16* and *ABCG26* are required for pollen wall integrity (Quilichini *et al.*, 2010; Wilson *et al.*, 2011). *AtABCGA1a* and *AtABCGA1b* are known to be involved in the early response to heat stress (HS) in Arabidopsis (Yadav *et al.*, 2014). The Arabidopsis *AtABCG7* transporter is involved in cuticle precursor trafficking (Buda *et al.*, 2013). *AtABCG25* is an exporter of ABA and is involved in the intercellular ABA signaling pathway (Kuromori *et al.*, 2010). The ABC transporter *ABCG1* is required for suberin formation in potato tuber periderm (Landgraf *et al.*, 2014).*ABCG15* and its Arabidopsis ortholog were shown to be required for pollen exine formation (Zhao *et al.*, 2015), and*ABCG32* functions in the formation of the developing leaf cuticle in Arabidopsis (Fabre *et al.*, 2016). However, the functions of some ABCG genes have not yet been fully identified.

We also compared the expression levels of 10 duplicated gene pairs in the pear ABCG gene family, and differences were detected between the two members of each gene pair. Although 80 ABCG transporters are present in Arabidopsis and pear, only 10 of them, *PbABCG4*, *PbABCG5*, *PbABCG6*, *PbABCG11*, *PbABCG14*, *PbABCG15*, *PbABCG20*, *PbABCG21*, *PbABCG32*, *PbABCG35*, gained our attention. Previous studies have shown that *PbABCG6*, *PbABCG20* may be involved in suberin formation. *PbABCG4* and *PbABCG5* were shown to be involved in the regulation of lipid-trafficking mechanisms. *ABCG32* is involved in the formation of the leaf cuticle and affects cutin composition and cuticle structure. Notably, *PbABCG6* and *PbABCG11* were highly expressed in all seven of the stages analyzed forthe two varieties of pears, especially in the exocarp of pear fruit, where suberin formation occurs. *PbABCG15* in rice participates in pollen exine development, and is used in the formation of the lipidic cuticle, and this may result in an increase in suberin in the exocarp also in pear fruit. *PbABCG15* expression dropped along the 75 days, possible because the decrease in lipid synthesis. *PbABCG6*, *PbABCG15* and *PbABCG20* were increasingly up-regulated over time, suggesting that these genes may play important roles in the process of pericarp browning in pear. However, further investigations will be required to determine the functions of *PbABCG* genes in pear. Some *PbABCG* genes showed unaltered or down-regulated expression over time, suggesting that these genes may operate in other signal transduction pathways in the complex regulatory network of the plant stress response. The results suggest that the duplicated genes exhibit significant functional divergence.

## Supplementary material

The following online material is available for this article:

Figure S1 - Visualization of the *PbABCG* genes mapped onto the different chromosomes in the pear genome.

Figure S2 - Localization and duplication of the *PbABCG* genes in the pear genome.

Figure S3 - The conserved motifs in the proteins.

Figure S4 - Exon structure.

Table S1 - Characteristics of *PbABCG* genes in pear.

Table S2 - Primer sequences.

Table S3 - Synteny and Ka/Ks related to genes in *PbABCG* genes in pear.

## References

[B1] Alejandro S, Lee Y, Tohge T, Sudre D, Osorio S, Park J, Bovet L, Lee Y, Geldner N, Fernie AR (2012). *AtABCG29* is a monolignol transporter involved in lignin
biosynthesis. Curr Biol.

[B2] Badri DV, Quintana N, El Kassis EG, Kim HK, Choi YH, Sugiyama A, Verpoorte R, Martinoia E, Manter DK, Vivanco JM (2009). An ABC transporter mutation alters root exudation of
phytochemicals that provoke an overhaul of natural soil
microbiota. Plant Physiol.

[B3] Buda GJ, Barnes WJ, Fich EA, Park S, Yeats TH, Zhao L, Domozych DS, Rose JK (2013). An ATP binding cassette transporter is required for cuticular wax
deposition and desiccation tolerance in the moss *Physcomitrella
patens*. Plant Cell.

[B4] Cai B, Yang X, Tuskan GA, Cheng ZM (2011). MicroSyn: A user friendly tool for detection of microsynteny in a
gene family. BMC Bioinformatics.

[B5] Du D, Cheng T, Pan H, Yang W, Wang J, Zhang Q (2013). Genome-wide identification, molecular evolution and expression
analyses of the phospholipase D gene family in three Rosaceae
species. Sci Hortic-Amsterdam.

[B6] Eddy SR (2011). Accelerated profile HMM searches. PLoS Comput Biol.

[B7] Edgar RC (2004). MUSCLE: a multiple sequence alignment method with reduced time
and space complexity. BMC Bioinformatics.

[B8] Fabre G, Garroum I, Mazurek S, Daraspe J, Mucciolo A, Sankar M, Humbel BM, Nawtath C (2016). The ABCG transporter
*PEC1*/*ABCG32* is required for the
formation of the developing leaf cuticle in *Arabidopsis*. New Phytol.

[B9] Fawcett JA, Maere S, Van de Peer Y (2009). Plants with double genomes might have had a better chance to
survive the Cretaceous-Tertiary extinction event. Proc Natl Acad Sci USA.

[B10] Heng W, Wang ZT, Jiang XH, Jia B, Liu P, Liu L, Ye ZF, Zhu LW (2016). The role of polyamines during exocarp formation in a russet
mutant of ‘Dangshansuli’ pear (*Pyrus bretchnederi
Rehd.*). Plant Cell Rep.

[B11] Higgins CF, Linton KJ (2004). The ATP switch model for ABC transporters. Nat Struct Mol Biol.

[B12] Hofmann NR (2014). Supply route: ABCG transporters act in the construction of
suberin barriers. Plant Cell.

[B13] Kang J, Hwang JU, Lee M, Kim YY, Assmann SM, Martinoia E, Lee Y (2011). PDR-type ABC transporter mediates cellular uptake of the
phytohormone abscisic acid. Proc Natl Acad Sci USA.

[B14] Kuromori T, Miyaji T, Yabuuchi H, Shimizu H, Sugimoto E, Kamiya A, Moriyama Y, Shinozaki K (2010). ABC transporter *AtABCG25* is involved in abscisic
acid transport and responses. Proc Natl Acad Sci USA.

[B15] Kuromori T, Sugimoto E, Shinozaki K (2011). *Arabidopsis* mutants of *AtABCG22*, an ABC
transporter gene, increase water transpiration and drought
susceptibility. Plant J.

[B16] Landgraf R, Smolka U, Altmann S, Eschen-Lippold L, Senning M, Sonnewald S, Weigel B, Frolova N, Strehmel N, Hause G (2014). The ABC transporter ABCG1 is required for suberin formation in
potato tuber periderm. Plant Cell.

[B17] McFarlane HE, Shin JJ, Bird DA, Samuels AL (2010). *Arabidopsis* ABCG transporters, which are required for
export of diverse cuticular lipids, dimerize in different
combinations. Plant Cell.

[B18] Nicolás MF, Barcellos FG, Nehab Hess P, Hungria M (2007). ABC transporters in *Mycoplasma hyopneumoniae* and
*Mycoplasma synoviae*: Insights into evolution and
pathogenicity. Genet Mol Biol.

[B19] Panikashvili D, Savaldi-Goldstein S, Mandel T, Yifhar T, Franke RB, Höfer R, Schreiber L, Chory J, Aharoni A (2007). The Arabidopsis
*DESPERADO*/*AtWBC11* transporter is
required for cutin and wax secretion. Plant Physiol.

[B20] Panikashvili D, Shi JX, Bocobza S, Franke RB, Schreiber L, Aharoni A (2010). The Arabidopsis DSO/ABCG11 transporter affects cutin metabolism
in reproductive organs and suberin in roots. Mol Plant.

[B21] Panikashvili D, Shi JX, Schreiber L, Aharoni A (2011). The Arabidopsis ABCG13 transporter is required for flower cuticle
secretion and patterning of the petal epidermis. New Phytol.

[B22] Qiao X, Li M, Li L, Yin H, Wu J, Zhang S (2015). Genome-wide identification and comparative analysis of the heat
shock transcription factor family in Chinese white pear (*Pyrus
bretschneideri*) and five other Rosaceae species. BMC Plant Biol.

[B23] Qin P, Tu B, Wang Y, Deng L, Quilichini TD, Li T, Wang H, Ma B, Li S (2013). ABCG15 encodes an ABC transporter protein, and is essential for
post-meiotic anther and pollen exine development in rice. Plant Cell Physiol.

[B24] Quilichini TD, Friedmann MC, Samuels AL, Douglas CJ (2010). ATP-binding cassette transporter G26 is required for male
fertility and pollen exine formation in Arabidopsis. Plant Physiol.

[B25] Saha J, Sengupta A, Gupta K, Gupta B (2015). Molecular phylogenetic study and expression analysis of
ATP-binding cassette transporter gene family in *Oryza
sativa* in response to salt stress. Comput Biol Chem.

[B26] Shiono K, Ando M, Nishiuchi S, Takahashi H, Watanabe K, Nakamura M, Matsuo Y, Yasuno N, Ymanouch U, Fujimoto M (2014). RCN1/OsABCG5, an ATP-binding cassette (ABC) transporter, is
required for hypodermal suberization of roots in rice (*Oryza
sativa*). Plant J.

[B27] Tamura K, Stecher G, Peterson D, Filipsk A, Kumar S (2013). MEGA6: Molecular Evolutionary Genetics Analysis version
6.0. Mol Biol Evol.

[B28] Verrier PJ, Bird D, Burla B, Dassa E, Forestier C, Geisler M, Klein M, Ukisaoglu UK, Lee Y, Martinoia E (2008). Plant ABC proteins - A unified nomenclature and updated
inventory. Trends Plant Sci.

[B29] Vishwanath SJ, Delude C, Domergue F, Rowland O (2015). Suberin: Biosynthesis, regulation, and polymer assembly of a
protective extracellular barrier. Plant Cell Rep.

[B30] Wang D, Zhang Y, Zhang Z, Zhu J, Yu J (2010). KaKs_Calculator 2.0: A toolkit incorporating gamma-series methods
and sliding window strategies. Genomics Proteomics Bioinfo.

[B31] Wilson ZA, Song J, Taylor B, Yang C (2011). The final split: The regulation of anther
dehiscence. J Exp Bot.

[B32] Wu J, Wang Z, Shi Z, Zhang S, Ming R, Zhu S, Chen NJ (2013). The genome of the pear (*Pyrus bretschneideri*
Rehd.). Genome Res.

[B33] Xi J, Xu P, Xiang CB (2012). Loss of AtPDR11, a plasma membrane-localized ABC transporter,
confers paraquat tolerance in *Arabidopsis thaliana*. Plant J.

[B34] Yadav V, Molina I, Ranathunge K, Castillo IQ, Rothstein SJ, Reed JW (2014). ABCG transporters are required for suberin and pollen wall
extracellular barriers in *Arabidopsis*. Plant Cell.

[B35] Zhao G, Shi J, Liang W, Xue F, Luo Q, Zhu L, Qu Z, Chen M, Schreiber L, Zhang D (2015). Two ATP binding cassette G transporters, rice ATP binding
cassette G26 and ATP binding cassette G15, collaboratively regulate rice
male reproduction. Plant Physiol.

