# Supplementary material for: Characterization and expression of the ABC family (G group) in ‘Dangshansuli’ pear (Pyrus bretschneideri Rehd.) and its russet mutant
Source: Genet Mol Biol. 2018 Jan-Mar;41(1):137–44. doi: 10.1590/1678-4685-GMB-2017-0109 (PMC5901498; doi:10.1590/1678-4685-GMB-2017-0109)
Supplement: Supplementary file 1 [file 1415-4757-GMB-41-01-2017-0109-s006.pdf]

Supplementary Material to "Characterization and expression of the ABC family (G group) in 'Dangshansuli' pear (*Pyrus bretschneideri* Rehd.) and its russet mutante"

Table S2 - Primer sequences for the ABCG genes in the exocarps of 'Dangshansuli' and russet mutant pear.

Primer names	Base sequence (5' to 3')
ABCG4-1R	TTCAACCTATTTCATTTAGTATTTATCGA
ABCG4-1F	AGGTGGACTAATATCTACATCCTGCTA
ABCG4-2F	GTTTTTCGTGTTCTATCGTGTGCT
ABCG4-2R	TTCATATTTGACTTTAGTTGACTACTTCTTT
ABCG5F	GGAGGTGTATGGTGACGGGA
ABCG5R	CATACAATTACTAGTTAAAGAAAAGGAGGT

Primer names	Base sequence (5' to 3')
ABCG6R	AGCAACAAGGCAAAGTAAAACAG
ABCG6F	AGCAGGGAGTGACGGACTTGAG
ABCG11F	GTCAATGCCTACCAAGCGAAAC
ABCG11R	ACATAAGGAGAAGCATTGGGGT
ABCG14F	GAATACCAACGGTGATGGACTG
ABCG14R	CTGATGATCGGTTGGCGTACT
ABCG15F	GCCGGTGTTTCAGTCACTCT
ABCG15R	TACTCAGTCCTGCAATGCCTC
ABCG20F	GACAACTCACCACTAGGAGCC
ABCG20R	AAGAACCCCAAAGCAATGGTGA
ABCG21F	TGGGAGTGCAATACTCGGTG
ABCG21R	GAGGTTGCCCCATCCTCAAT

Primer names	Base sequence (5' to 3')
ABCG32F	TAGCCGGCATTATGGTGGTC
ABCG32R	CCCAGAACTGAAGCAATGTGG
ABCG35F	GATATGTCCCGTGGCATGGA
ABCG35R	TGAAGGCGAAGAAGACTCCG
