# Supplementary material for: Characterization and expression of the ABC family (G group) in ‘Dangshansuli’ pear (Pyrus bretschneideri Rehd.) and its russet mutant
Source: Genet Mol Biol. 2018 Jan-Mar;41(1):137–44. doi: 10.1590/1678-4685-GMB-2017-0109 (PMC5901498; doi:10.1590/1678-4685-GMB-2017-0109)
Supplement: Supplementary file 2 [file 1415-4757-GMB-41-01-2017-0109-s005.pdf]

Supplementary Material to "Characterization and expression of the ABC family (G group) in 'Dangshansuli' pear (*Pyrus bretschneideri* Rehd.) and its russet mutante"

Table S1 - Characteristics of ABCG genes in 'Dangshansuli'pear.

Gene ID*[1]	Gene ID*[2]	Gene ID*[3]	Chromosome	Start	End	Strand	Amino acid length	Molecular mass values	Isoelectric point
PbABCG16a	Pbr026485	LOC103928588	Chr7	9962090	9964617	+	751	82.60827	9.511
PbABCG16b	Pbr032285	LOC103948344	Chr7	10296939	10299809	-	751	82.92444	9.1938
PbABCG16c	Pbr038144	LOC103936749	scaffold797.0	78079	81063	-	751	82.79962	9.6043
PbABCG2a	Pbr008253	LOC103933813	Chr12	9725098	9727923	-	729	81.07879	8.8324
PbABCG2b	Pbr032212	LOC103932967	Chr8	3097337	3099969	-	727	81.02604	9.7471
PbABCG19b	Pbr028364	LOC103946918	Chr6	2365307	2368093	+	820	92.29635	9.4259
PbABCG19a	Pbr038838	LOC103950663	Chr14	16706307	16709072	+	809	91.02887	9.6304
PbABCG17c	Pbr040113	LOC103937641	scaffold872.0	128804	131654	-	743	82.88499	9.6111
PbABCG17a	Pbr004664	LOC103954689	Chr15	21011865	21015202	+	741	82.59777	9.7771
PbABCG17b	Pbr013994	-	scaffold206.0.1	111796	115133	-	741	82.59272	9.7275
PbABCG8b	Pbr021544	LOC103966156	scaffold320.0	263873	265681	-	602	66.9032	9.4768
PbABCG8c	Pbr021545	LOC103966157	scaffold320.0	307821	309629	+	602	66.9032	9.4768
PbABCG8a	Pbr010836	LOC103958197	Chr13	450434	452500	+	601	66.96141	9.6175
PbABCG5	Pbr027932	LOC103946773	Chr15	10596952	10599306	+	672	74.8078	8.4667

Gene ID*[1]	Gene ID*[2]	Gene ID*[3]	Chromosome	Start	End	Strand	Amino acid length	Molecular mass values	Isoelectric point
PbABCG10c	Pbr038226	LOC103932334	Chr11	4353494	4355320	-	608	68.14403	9.5386
PbABCG10a	Pbr025834	LOC103927933	Chr3	1903836	1905662	-	608	68.23977	8.9369
PbABCG10b	Pbr034373	LOC103934278	scaffold640.0	102783	104609	-	608	68.35698	8.9604
PbABCG25b	Pbr018258	LOC103964120	Chr10	24137240	24138392	-	324	35.11167	8.6725
PbABCG25a	Pbr018263	LOC103964121	Chr10	24229617	24236942	+	654	72.20918	8.8721
PbABCG25c	Pbr040133	LOC103937713	Chr10	23562926	23565139	+	331	35.91257	9.0149
PbABCG21a	Pbr013231	LOC103946921	Chr3	21585996	21589869	+	671	74.92667	8.4923
PbABCG21b	Pbr027509	LOC103929216	Chr13	2614521	2618538	+	715	79.27768	9.0629
PbABCG9	Pbr022331	LOC103966992	Chr11	5988460	5991656	-	622	69.06952	9.9739
PbABCG26	Pbr008669	LOC103956915	Chr17	5852280	5855641	-	702	79.85684	8.5218
PbABCG22c	Pbr039401	LOC103951205	Chr10	20455656	20459471	+	768	85.45994	9.4418
PbABCG22a	Pbr001309	LOC103952710	Chr12	18083454	18089510	+	747	82.54539	7.425
PbABCG22b	Pbr010296	LOC103940192	Chr14	1630091	1637121	+	747	82.5666	9.0645
PbABCG3a	Pbr019006	LOC103964822	Chr3	15369227	15374741	+	711	78.69554	9.1112
PbABCG3b	Pbr030340	LOC103947696	Chr11	29988477	29993847	+	711	78.74341	8.5371
PbABCG11a	Pbr028447	LOC103930284	scaffold471.0.1	112346	117172	+	686	75.77933	8.2085
PbABCG11b	Pbr037888	LOC103936479	Chr5	1691307	1696372	-	702	77.52573	8.875
PbABCG15a	Pbr002037	LOC103953191	Chr14	7707051	7712257	+	677	75.2243	8.6908
PbABCG15f	Pbr030433	LOC103931653	Chr10	15446359	15451172	+	689	76.16238	8.6763
PbABCG15e	Pbr017197	LOC103963798	Chr3	8744863	8750399	+	703	78.59418	9.4782
PbABCG15d	Pbr017196	LOC103963796	Chr3	8724218	8728740	-	686	76.17321	9.2774
PbABCG15b	Pbr006928	LOC103955899	Chr11	10009835	10017362	-	659	73.47338	9.1699
PbABCG15c	Pbr007063	LOC103942099	scaffold1396.0	2541	10059	-	598	67.2274	9.2846
PbABCG24	Pbr010690	LOC103958377	Chr1	5157531	5162913	+	1110	122.78105	8.3614
PbNAP12	Pbr020543	LOC103965465	Chr12	8021418	8029147	-	1115	124.02108	9.3218
PbABCG28c	Pbr030327	LOC103947686	Chr11	30144933	30156266	+	1068	118.0247	9.1037

Gene ID*[1]	Gene ID*[2]	Gene ID*[3]	Chromosome	Start	End	Strand	Amino acid length	Molecular mass values	Isoelectric point
PbABCG28a	Pbr008372	LOC103956733	Chr3	16170117	16175423	-	814	90.16358	9.6404
PbABCG28b	Pbr018986	LOC103964811	Chr3	15105291	15115120	-	938	103.92426	9.4063
PbABCG7	Pbr028734	LOC103947048	Chr13	1517381	1521945	+	729	79.42906	6.7224
PbABCG39a	Pbr001652	-	Chr8	6543498	6546626	-	650	74.67133	7.292
PbABCG39c	Pbr009350	LOC103942676	scaffold1599.0	18512	20623	+	346	38.50228	7.3033
PbABCG34j	Pbr017806	LOC103963646	Chr12	20658693	20666315	+	1483	167.51098	7.8756
PbABCG34f	Pbr005538	LOC103955219	Chr12	21459427	21466570	-	1452	164.01298	7.7856
PbABCG34e	Pbr005535	LOC103955214	Chr12	21404319	21411462	-	1452	163.98493	7.6774
PbABCG34d	Pbr005533	LOC103955211	Chr12	21348046	21356404	-	1452	164.47316	8.0471
PbABCG34h	Pbr016785	LOC103944282	Chr14	3040552	3050484	+	1741	196.93989	8.9798
PbABCG34i	Pbr016786	LOC103944283	Chr14	3051645	3058330	+	1436	162.821	7.4166
PbABCG39b	Pbr028690	LOC103947005	Chr13	1115167	1122193	-	1435	162.74258	7.1314
PbABCG34b	Pbr004829	LOC103954767	Chr3	13333149	13340368	-	1439	163.08598	7.8988
PbABCG34a	Pbr001651	LOC103939731	Chr8	6533506	6540345	+	1439	163.09301	8.1943
PbABCG34c	Pbr004830	LOC103954769	Chr3	13361557	13369970	-	1448	164.54954	8.6291
PbABCG34g	Pbr009349	LOC103942675	scaffold1599.0	7443	14208	+	1412	160.18972	6.6754
PbABCG40b	Pbr003177	LOC103953954	Chr2	22050550	22056876	+	1387	155.80633	7.6464
PbABCG40h	Pbr038066	-	scaffold793.0	68780	75079	-	1387	155.70927	7.6463
PbABCG40d	Pbr025435	LOC103927579	Chr15	389353	396711	+	1424	160.90258	7.4162
PbABCG40a	Pbr001472	LOC103939592	scaffold1034.0	16920	23335	+	1313	147.77555	7.8954
PbABCG40i	Pbr003545	-	scaffold1154.0	10383	11466	+	164	18.0208	6.2408
PbABCG40c	Pbr025434	LOC103927580	Chr15	376796	384077	+	1489	167.95495	6.9393
PbABCG40g	Pbr036064	LOC103935388	Chr9	11789544	11796953	+	1403	158.70627	8.286
PbABCG40e	Pbr025876	LOC103927975	Chr10	11022941	11029493	-	1433	161.78225	8.2975
PbABCG40f	Pbr032922	LOC103959952	Chr5	16893516	16900391	-	1437	162.51382	8.207
PbABCG32a	Pbr014071	LOC103960664	Chr2	19194068	19201662	-	1419	161.47333	8.14

Gene ID*[1]	Gene ID*[2]	Gene ID*[3]	Chromosome	Start	End	Strand	Amino acid length	Molecular mass values	Isoelectric point
PbABCG32b	Pbr016988	LOC103962994	Chr15	14404542	14411931	-	1419	161.86876	8.2874
PbABCG31a	Pbr000915	LOC103939227	scaffold1001.0	56375	65331	-	1425	159.36746	8.9576
PbABCG31b	Pbr014244	LOC103960748	Chr11	17037831	17045112	-	1169	130.49761	8.6441
PbABCG37c	Pbr037759	LOC103950309	Chr16	5685119	5693451	+	1453	162.73119	7.5095
PbABCG37a	Pbr006706	LOC103955857	Chr4	2507891	2514551	+	1447	163.21637	7.7184
PbABCG30	Pbr018550	-	Chr13	7536596	7543357	+	1121	126.65135	7.2904
PbABCG37b	Pbr037758	LOC103950308	Chr16	5694535	5701031	-	1312	147.92073	8.3915
PbABCG29a	Pbr019117	LOC103944887	Chr9	11553815	11563586	+	1491	168.71149	9.1999
PbABCG29b	Pbr034728	LOC103934504	Chr9	8987632	8997462	+	1491	168.7225	9.0899
PbABCG36c	Pbr019121	LOC103944819	Chr9	11493113	11501097	+	1315	148.11402	8.4988
PbABCG36a	Pbr002804	LOC103940188	Chr17	5147340	5156962	-	1497	169.14559	8.8634
PbABCG36b	Pbr019119	LOC103944820	Chr9	11518741	11528189	+	1477	166.67536	8.0263
PbABCG41	Pbr019123	LOC103944818	Chr9	11450160	11459482	+	1346	152.6126	8.6243
PbABCG36d	Pbr019124	LOC103944817	Chr9	11416121	11426132	+	1497	169.28749	7.9394

*[1]: Gene nameL; *[2]: Gene ID: Pbr_v1.0 (http://peargenome.njau.edu.cn); *[3]: Gene locus in NCBI ; MW: molecular weight; IP: Isoelectric point; *E*-value: *E*-value of InterProSca
