# Supplementary material for: Characterization and expression of the ABC family (G group) in ‘Dangshansuli’ pear (Pyrus bretschneideri Rehd.) and its russet mutant
Source: Genet Mol Biol. 2018 Jan-Mar;41(1):137–44. doi: 10.1590/1678-4685-GMB-2017-0109 (PMC5901498; doi:10.1590/1678-4685-GMB-2017-0109)
Supplement: Supplementary file 4 [file 1415-4757-GMB-41-01-2017-0109-s002.pdf]

Supplementary Material to "Characterization and expression of the ABC family (G group) in 'Dangshansuli' pear (*Pyrus bretschneideri* Rehd.) and its russet mutante"

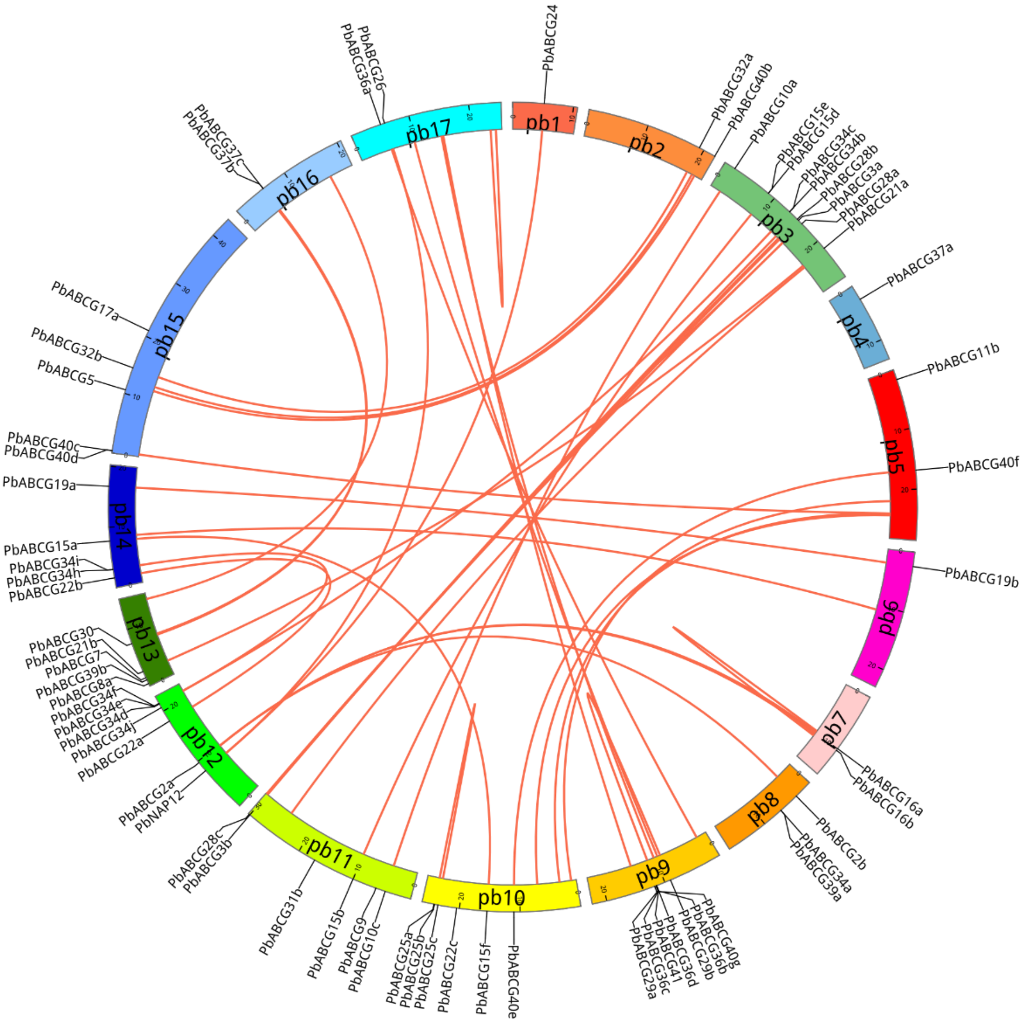

Figure S2 - Localization and duplication of the *PbABCG* genes in the pear genome. Circular visualization of the *PbABCG* genes was mapped on the different chromosomes in the genome using the Circos software. Chromosome number is indicated on the chromosome. The synteny relationship between each pair of *PbABCG* genes were detected by using the MicroSyn software. The genes have synteny relationship are linked by lines. Red link: >30 anchors in a synteny block, blue link: 20-30 anchors, green link: 10-20 anchors, gray link: 5-10 anchors.
