# Supplementary material for: Characterization and expression of the ABC family (G group) in ‘Dangshansuli’ pear (Pyrus bretschneideri Rehd.) and its russet mutant
Source: Genet Mol Biol. 2018 Jan-Mar;41(1):137–44. doi: 10.1590/1678-4685-GMB-2017-0109 (PMC5901498; doi:10.1590/1678-4685-GMB-2017-0109)

Supplementary Material to "Characterization and expression of the ABC family (G group) in 'Dangshansuli' pear (*Pyrus bretschneideri* Rehd.) and its russet mutante"

Figure S3 - The conserved motifs in the proteins. The conserved motifs in the proteins were detected by MEME (http://meme.nbcr.net/meme/cgibin/meme.cgi) with the following parameters: number of repetitions: any; maximum number of motifs: 50and the optimum motif widths: 6-100 amino acid residues. Corresponding motifs also detested using programs in InterProScan. Names of genes are indicated on the left. Lines represent protein regions without detected motif. Different motifs are highlighted with different colored boxes with numbers 1 to 20, which represent the motif identifier in file meme.txt.

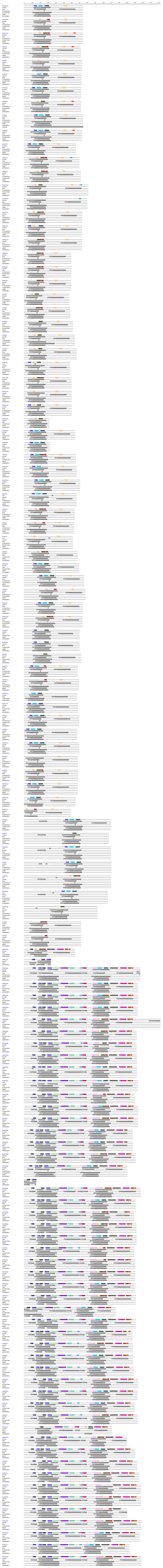

Supplement: Supplementary file 5 [file 1415-4757-GMB-41-01-2017-0109-s003.pdf]
