# Supplementary material for: Characterization and expression of the ABC family (G group) in ‘Dangshansuli’ pear (Pyrus bretschneideri Rehd.) and its russet mutant
Source: Genet Mol Biol. 2018 Jan-Mar;41(1):137–44. doi: 10.1590/1678-4685-GMB-2017-0109 (PMC5901498; doi:10.1590/1678-4685-GMB-2017-0109)
Supplement: Supplementary file 6 [file 1415-4757-GMB-41-01-2017-0109-s004.pdf]

Supplementary Material to "Characterization and expression of the ABC family (G group) in 'Dangshansuli' pear (*Pyrus bretschneideri* Rehd.) and its russet mutante"

Figure S4 - Exons, represented by boxes, were drawn to scale. Lines connecting two exons represent an intron. PF00005 domain (ATP-binding domain of ABC transporters) is marked in red. Intron phases 0, 1 and 2 are indicated by numbers 0, 1 and 2, respectively.

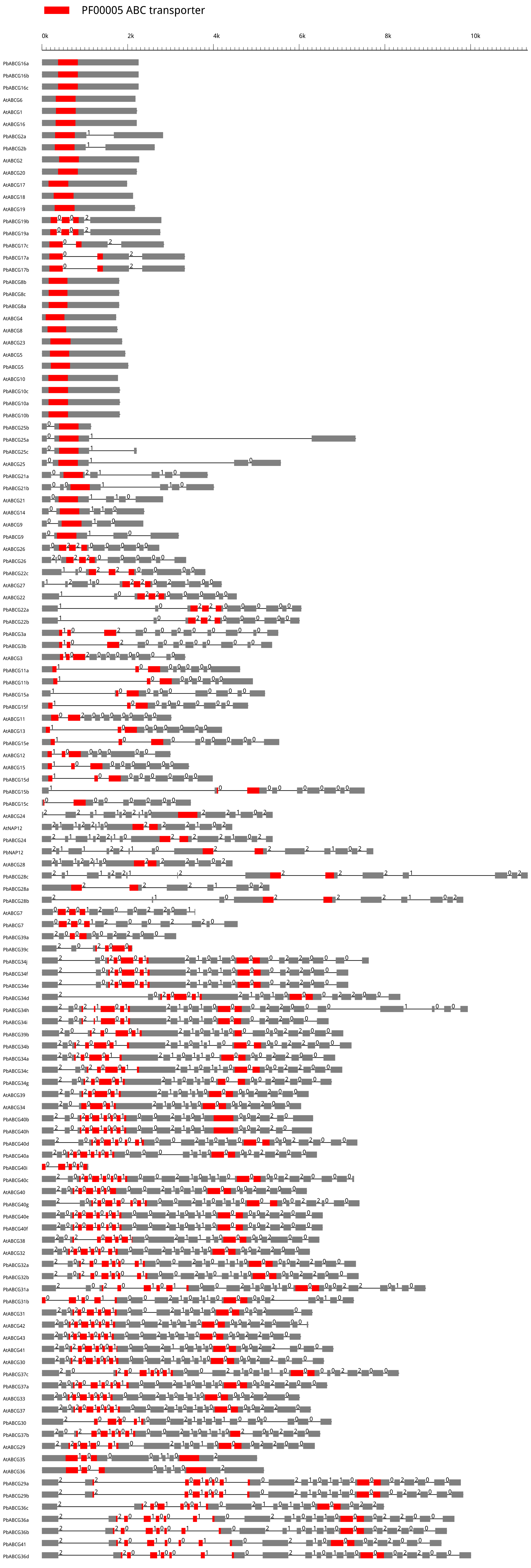
PF00005 ABC transporter
0k
2k
4k
6k
8k
10k
PbABCG16a
PbABCG16b
PbABCG16c
AtABCG6
AtABCG1
AtABCG16
PbABCG2a
PbABCG2b
AtABCG2
AtABCG20
AtABCG17
AtABCG18
AtABCG19
PbABCG19b
PbABCG19a
PbABCG17c
PbABCG17a
PbABCG17b
PbABCG8b
PbABCG8c
PbABCG8a
AtABCG4
AtABCG8
AtABCG23
AtABCG5
PbABCG5
AtABCG10
PbABCG10c
PbABCG10a
PbABCG10b
PbABCG25b
PbABCG25a
PbABCG25c
AtABCG25
PbABCG21a
PbABCG21b
AtABCG21
AtABCG14
AtABCG9
PbABCG9
AtABCG26
PbABCG26
PbABCG22c
AtABCG27
AtABCG22
PbABCG22a
PbABCG22b
PbABCG3a
PbABCG3b
AtABCG3
PbABCG11a
PbABCG11b
PbABCG15a
PbABCG15f
AtABCG11
AtABCG13
PbABCG15e
AtABCG12
AtABCG15
PbABCG15d
PbABCG15b
PbABCG15c
AtABCG24
AtNAP12
PbABCG24
PbNAP12
AtABCG28
PbABCG28c
PbABCG28a
PbABCG28b
AtABCG7
PbABCG7
PbABCG39a
PbABCG39c
PbABCG34j
PbABCG34f
PbABCG34e
PbABCG34d
PbABCG34h
PbABCG34i
PbABCG39b
PbABCG34b
PbABCG34a
PbABCG34c
PbABCG34g
AtABCG39
AtABCG34
PbABCG40b
PbABCG40h
PbABCG40d
PbABCG40a
PbABCG40i
PbABCG40c
AtABCG40
PbABCG40g
PbABCG40e
PbABCG40f
AtABCG38
AtABCG32
PbABCG32a
PbABCG32b
PbABCG31a
PbABCG31b
AtABCG31
AtABCG42
AtABCG43
AtABCG41
AtABCG30
PbABCG37c
PbABCG37a
AtABCG33
AtABCG37
PbABCG30
PbABCG37b
AtABCG29
AtABCG35
AtABCG36
PbABCG29a
PbABCG29b
PbABCG36c
PbABCG36a
PbABCG36b
PbABCG41
PbABCG36d
