# Supplementary material for: Characterization and expression of the ABC family (G group) in ‘Dangshansuli’ pear (Pyrus bretschneideri Rehd.) and its russet mutant
Source: Genet Mol Biol. 2018 Jan-Mar;41(1):137–44. doi: 10.1590/1678-4685-GMB-2017-0109 (PMC5901498; doi:10.1590/1678-4685-GMB-2017-0109)
Supplement: Supplementary file 7 [file 1415-4757-GMB-41-01-2017-0109-s007.pdf]

Supplementary Material to "Characterization and expression of the ABC family (G group) in 'Dangshansuli' pear (*Pyrus bretschneideri* Rehd.) and its russet mutante"

Table S3- Synteny and Ka/Ks related to genes in ABC G subfamily in 'Dangshansuli' pear.

Pair of gene	Anchors	*E* value	Ka	Ks	Ka/Ks
PbABCG19b vs PbABCG19a	47	1.96E-093	0.04	0.2	0.22
PbABCG32a vs PbABCG32b	40	1.32E-076	0.04	0.16	0.23
PbABCG21a vs PbABCG21b	32	2.84E-066	0.06	0.19	0.3
PbABCG34h vs PbABCG34d	30	5.52E-060	0.06	0.2	0.28
PbABCG22b vs PbABCG22a	24	1.69E-051	0.04	0.15	0.29
PbABCG8a vs PbABCG8b	23	4.54E-041	0.04	0.22	0.17
PbABCG16b vs PbABCG16c	16	7.09E-025	0.03	0.43	0.07
PbABCG31a vs PbABCG31b	15	6.69E-027	0	0.01	0.17
PbABCG25c vs PbABCG25b	15	2.08E-029	0.03	0.05	0.7
PbABCG16a vs PbABCG16c	14	1.71E-019	0.03	0.42	0.07
PbABCG40f vs PbABCG40e	13	6.32E-028	0.04	0.14	0.26
PbABCG10a vs PbABCG10c	11	1.85E-024	0.06	0.14	0.41
PbABCG10c vs PbABCG10b	10	4.11E-019	0.06	0.15	0.36
PbABCG3a vs PbABCG3b	9	5.18E-013	0.03	0.15	0.23

Pair of gene	Anchors	*E* value	Ka	Ks	Ka/Ks
PbABCG11b vs PbABCG11a	9	5.37E-017	0.05	0.16	0.3
PbABCG29b vs PbABCG36d	9	1.09E-022	0.13	0.48	0.26
PbABCG37c vs PbABCG30	9	3.70E-018	0.33	1.63	0.2
PbABCG16a vs PbABCG2a	8	4.12E-012	0.2	4.64	0.04
PbABCG16b vs PbABCG2a	8	2.86E-011	0.2	4.65	0.04
PbABCG2b vs PbABCG2a	7	1.06E-015	0.05	0.17	0.27
PbABCG15d vs PbABCG15b	6	1.05E-009	0.04	0.19	0.21
PbABCG28b vs PbABCG28c	6	1.27E-008	0.08	0.21	0.39
PbABCG28a vs PbABCG28c	5	2.76E-008	0.05	0.17	0.31
PbABCG28b vs PbABCG28a	4	2.05E-008	0	0	2.38
PbABCG17b vs PbABCG17a	4	1.21E-007	0.01	0.01	0.51
PbABCG15a vs PbABCG15f	4	3.65E-008	0.06	0.26	0.25
PbABCG24 vs PbNAP12	3	1.14E-004	0.27	2.19	0.12
PbABCG10a vs PbABCG10b	3	1.60E-005	0.01	0.01	0.78
PbABCG34b vs PbABCG34d	3	2.17E-003	0.16	1.33	0.12
PbABCG26 vs PbNAP12	3	4.66E-005	1.04	3.16	0.33
PbABCG15b vs PbABCG15c	3	9.80E-005	0	0.01	0.32
PbABCG2a vs PbABCG16c	3	1.78E-004	0.19	4.62	0.04
